# Upper extremity rehabilitation of stroke: Facilitation of corticospinal excitability using virtual mirror paradigm

**DOI:** 10.1186/1743-0003-9-71

**Published:** 2012-10-04

**Authors:** Youn Joo Kang, Hae Kyung Park, Hyun Jung Kim, Taeo Lim, Jeonghun Ku, Sangwoo Cho, Sun I Kim, Eun Sook Park

**Affiliations:** 1 Department of Rehabilitation Medicine, Eulji Hospital, Eulji University School of Medicine Seoul, Seoul, Korea; 2 Department of Physical Medicine and Rehabilitation, Graduate School, Yonsei University, Seoul, Korea; 3 Department of Biomedical Engineering, Keimyung University, Daegu, Korea; 4 Department of Biomedical Engineering, Hanyang University, Seoul, Korea; 5 Medical Device Development Center, Osong Medical Innovation Foundation, Chungbuk, Korea

**Keywords:** Stroke, Corticospinal excitability, Transcranial magnetic stimulation, Virtual reality, Feedback

## Abstract

**Background:**

Several experimental studies in stroke patients suggest that mirror therapy and various virtual reality programs facilitate motor rehabilitation. However, the underlying mechanisms for these therapeutic effects have not been previously described.

**Objectives:**

We attempted to delineate the changes in corticospinal excitability when individuals were asked to exercise their upper extremity using a real mirror and virtual mirror. Moreover, we attempted to delineate the role of visual modulation within the virtual environment that affected corticospinal excitability in healthy subjects and stroke patients.

**Methods:**

A total of 18 healthy subjects and 18 hemiplegic patients were enrolled into the study. Motor evoked potential (MEP)s from transcranial magnetic stimulation were recorded in the flexor carpi radialis of the non-dominant or affected upper extremity using three different conditions: (A) relaxation; (B) real mirror; and (C) virtual mirror. Moreover, we compared the MEPs from the virtual mirror paradigm using continuous visual feedback or intermittent visual feedback.

**Results:**

The rates of amplitude increment and latency decrement of MEPs in both groups were higher during the virtual mirror task than during the real mirror. In healthy subjects and stroke patients, the virtual mirror task with intermittent visual feedback significantly facilitated corticospinal excitability of MEPs compared with continuous visual feedback.

**Conclusion:**

Corticospinal excitability was facilitated to a greater extent in the virtual mirror paradigm than in the real mirror and in intermittent visual feedback than in the continuous visual feedback, in both groups. This provides neurophysiological evidence supporting the application of the virtual mirror paradigm using various visual modulation technologies to upper extremity rehabilitation in stroke patients.

## Background

The incidence of stroke is growing, and there is more than 50% of stroke patients suffer from upper extremity disabilities, which can cause the stroke patient to trouble in activities of daily living [1]. In addition, after a stroke, more than 50% of patients report continuous disability of upper extremity function: even after conventional treatment, and learned nonuse—the avoidance of the use of the injured arm—is observed frequently [2]. For these reasons, programs aimed at restoring the function of upper extremities are an important part of stroke rehabilitation.

New treatment methods for upper extremity rehabilitation, based on the motor learning theory, are being assessed. Representative treatment methods that have been emerging recently include the constraint-induced movement theory, robot-arm training, training using virtual reality (VR), mental practice, and mirror therapy [3]. Even though results supporting the effectiveness of these methods are scarce, a wide range of randomized controlled clinical trials have been conducted [4].

Mirror therapy provides effective treatment for phantom limb pain in amputation patients [5]. Many studies have applied this therapy to hemiplegic patients, to investigate and validate its clinical effects based on the hypothesis that the visual illusion evoked by the mirror reflection of the unaffected arm, while blocking the visualization of the affected arm, would improve the motor abilities of these patients [6,7]. The exercise therapy paradigm using a mirror can be installed and used easily, thus being applicable in any place and providing patients with an opportunity to practice repeatedly. However, some disadvantages of this method have been noticed, based on reduced clinical compliance for stroke patients.

VR has become more important in the rehabilitation of stroke patients, based on its advantages: it provides patients with a more realistic, varied, and enhanced sensory perception and facilitates motor learning based on various feedback mechanisms [8]. However, insufficient studies have been performed on VR-based rehabilitation programs using randomly controlled cases [9] and such studies are highly desirable to clarify the synergistic effect that is usually provided by other conventional therapies.

According to recent transcranial magnetic stimulation (TMS) and functional magnetic resonance image (fMRI) research, the primary motor cortex can reorganize and modulate the interactions between the ipsilesional and contralesional motor cortex following a stroke [10,11]. Abnormal strong interhemispheric inhibition from the contralesional to ipsilesional motor cortex was observed in stroke survivors, which was associated with a poor functional outcome and ipsilesional motor cortical activation, is important for good motor recovery [10-12]. This indicates that ipsilesional motor cortical priming in stroke considered an important part of the management of the balance between hemispheres and of the recovery of functions. The method of voluntary exercise evokes the strongest facilitation at the cortex and spine levels. However, an alternative facilitation method is necessary because voluntary movements are difficult for stroke patients with severe paralysis of affected limbs. Therefore, the new treatment paradigm, which induced ipsilesional motor cortical priming such as mirror therapy combined with VR, was meaningful in clinical setting.

The virtual mirror exercise paradigm was designed specifically for this study and its effectiveness was investigated by comparing the MEPs evoked by the real mirror therapy paradigm. Furthermore, we investigated whether controlling for visual feedback to motion in the virtual mirror exercise system affected corticospinal activation, which could improve the effectiveness of motor learning. For this purpose, we used single-pulse transcranial magnetic stimulation (TMS). The amplitude and latency of MEPs were derived from the target muscle. TMS was advantageous in allowing the evaluation of *in vivo* anatomical cortico-cortical connectivity, and identifying corticospinal excitability according to various experimental conditions on real time basis [13].

## Methods

### Participants

Eighteen healthy Right-handed subjects and 18 stroke patients were recruited for the experiment. The mean age of the two groups was 30.9 ± 2.22 years in the healthy subjects and 61.33 ± 11.59 years in the stroke patients. Previous studies revealed that age did not seem to be a significant contributor to variations in MEP amplitude [14,15]. Therefore, we did not match the mean age of the two groups.

The healthy volunteers had no history of neurological disease, and no abnormalities were observed in musculoskeletal, neurological, or physical examinations. The stroke patients; (1) had suffered a primary ischemic or hemorrhagic stroke as revealed by computed tomography (CT) or magnetic resonance imaging (MRI) scan; (2) presented with mild to moderate paresis of the upper extremity (upper extremity manual muscle function test > grade 3); (3) lacked any additional neurological disease causing a motor deficit; and (4) showed no severe deficits in communication, memory, or orientation, with a Mini-Mental State Examination score > 24. The causes of stroke were infarction in 13 patients and hemorrhage in five patients; 12 patients had a right-sided lesion and six patients had a left-sided lesion (Table 1). Participants that had a pacemaker, contraindication to magnetic stimulation (such as seizure history), severe depression, apraxia, possible pregnancy, or were pregnant were excluded. All participants that consented to participate in this study were informed about TMS and the experimental protocol, which were approved by the institutional review board of our Hospital.

**Table 1:** Demographic and clinical characteristics of stroke patients

**Patient no.**	**Sex**	**Age(years)**	**Weeks since infarction**	**Etiology**	**Site of lesion**	**Functional independence measure**
1	M	64	5	Infarction	Rt. Th (subcortical)	96
2	F	45	12	Infarction	Rt. BG (subcortical)	116
3	F	50	9	Hemorrhage	Rt. BG (subcortical)	112
4	F	77	20	Infarction	Rt. pontine	87
5	M	67	6	Infarction	Lt. Th (subcortical)	98
6	M	50	47	Infarction	Lt. Th (subcortical)	120
7	F	54	96	Infarction	Rt. BG (subcortical)	126
8	M	63	20	Hemorrhage	Rt. Th (subcortical)	114
9	M	61	5	Infarction	Rt. BG (subcortical)	90
10	M	69	7	Hemorrhage	Lt. P,O (cortical and subcortical)	100
11	M	72	6	Infarction	Lt. BG, Th (subcortical)	92
12	M	72	16	Infarction	Lt. CR (cortical and subcortical)	76
13	M	50	40	Hemorrhage	Rt. F, P (subcortical)	91
14	M	71	5	Infarction	Rt. BG, Th (subcortical)	86
15	F	65	8	Infarction	Rt. BG (subcortical)	100
16	M	70	6	Infarction	Rt. F,O (cortical and subcortical)	92
17	M	34	24	Hemorrhage	Rt. cerebellum	112
18	F	70	22	Infarction	Lt. CR (cortical and subcortical)	91

Rt., right; Lt., left; BG, basal ganglia; Th, thalamus; F, frontal lobe; P, parietal lobe; O, occipital lobe; MCA, middle cerebral artery; CR, corona radiata.

### Transcranial magnetic stimulation

TMS was achieved using the figure-eight coil by MagVenture®MagPro (MagVenture, Lucernemarken, Denmark). Electromyography (EMG) signals were measured using the EMG system AlpinebioMed® Keypoint (Fountain Valley, CA, USA). To localize the target for stimulation, each subject wore a hat marked with 3 cm squares; the center of the hat was positioned to the bisection line of the nasion and inion/ear-to-ear (Cz) while sitting comfortably with both hands placed on a table. TMS was applied to the nondominant (left) hemisphere in healthy subjects and to the affected hemisphere in stroke patients. The electrodes used to measure MEPs in healthy subjects were attached to the motor point of the flexor carpi radialis (FCR) of the left upper extremity for the active recording, and to the tendon of the corresponding muscle for reference. In stroke patients, MEPs were acquired from the same muscle that was assessed in the affected upper extremity. The motor threshold was defined as the lowest stimulus intensity sufficient to elicit six MEPs of >50μV in a series of ten stimuli [16]. Stimulus intensity was adjusted at 120% of motor threshold.

### Experimental protocol

**Figure 1:**
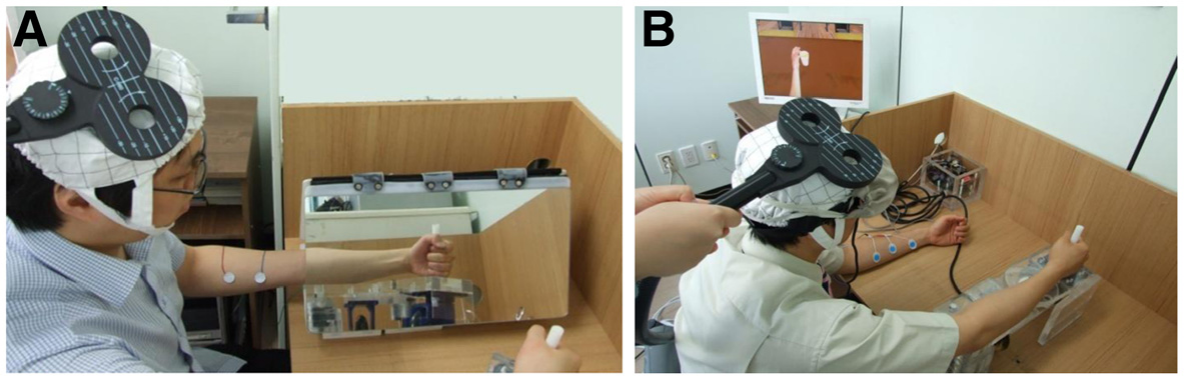
**Setup of the real mirror (A) and virtual mirror (B) experiments.** In the virtual mirror task, the subjects wore a head-mounted display while sitting in front of a table. The left upper extremity of healthy subjects, or the unaffected upper extremity of stroke patients, was placed on the experimental device, which records the angle of the wrist movements. Subjects could see a virtual cup on a virtual table and a virtual upper extremity contralateral to the moving upper extremity through the head-mounted display. The movements of the virtual upper extremity were controlled by the movements of the real moving upper extremity.

Subjects performed two experimental trials. All subjects randomly performed three assigned tasks to eliminate the order effect, relaxation, real mirror and virtual mirror task in experiment I and relaxation, continuous feedback and intermittent feedback task in experiment II. Between each task, at least three minutes rest was provided. In the relaxation condition, subjects were told to close their eyes and relax, and the left upper extremity in healthy subjects, or the affected upper extremity in the stroke group, was completely relaxed, which was confirmed using the EMG signal. In other tasks except relaxation, subjects were asked to perform a repetitive wrist flexion-extension exercise (0-60°) at 1 Hz, as was given by metronome. A goniometer was used to measure the angle of wrist joint. Through the experiments, 20 MEPs were obtained and their amplitude and latency values were averaged. TMS was delivered in the flexion phase of wrist movement corresponding to a wrist joint angle of 5° during every fifth flexion-extension cycle (inter-stimulus interval of 10s) [17]. To minimize the contraction of the muscles other than the wrist, the shoulder and elbow joint of moving upper extremity were fixed under the movement plate throughout the experiment (Figure 1). Subjects were also instructed to concentrate on observing their wrist movement in mirror or virtual environment and not to move their left or affected upper extremity and maintain relaxation. Background EMG activities of FCR muscle of non-moving upper extremity was carefully monitored in each MEP recording. If any background MEP activities were detected, the associated MEPs were omitted from the analysis.

### Experiment I(real mirror vs virtual mirror task)

#### Real mirror task

Subjects were asked to perform a wrist flexion-extension exercise at a constant pace of 1 Hz, using the unaffected upper extremity for the stroke patients or the right upper extremity for the healthy subjects, with a mirror box designed in our department (Figure 1). For this exercise, the affected or left (non- moving) upper extremity of subject was placed and relaxed in the mirror box positioned at the center of the table. The contralateral unaffected or right (moving) upper extremity of subjects was placed on the table. The mirror box was positioned so that the reflection would appear to be the non-moving upper extremity. Subjects were asked to observe and concentrate on the reflected image movement while performing a wrist flexion-extension exercise (0-60°) at 1 Hz using the right or unaffected upper extremity.

#### Virtual mirror task

The virtual mirror task was designed by the clinicians, biomedical engineers, and occupational therapist of our team. The system was developed by a joined team of researchers from Eulji University, Keimyung Univeristy and Hanyang Universities of South Korea. The hardware was comprised of a personal computer equipped with a head-mounted display (HMD; z800 3DVior, eMagin Co., Bellevue, WA, USA) and angle detector: encoders (EP50S series, Autonics Co., Yangsan-si, Gyeongsangnam-do, KOREA) implemented with hinges and plates with wheels to detect upper extremity movements. The virtual environment was provided in monoscopic mode with a resolution of 800×600 pixels through the HMD. In the virtual mirror experiment setting, the visual field was blocked so that subjects could see the virtual cup on a virtual table, and a virtual upper extremity only through the HMD. Subjects could see a virtual contralateral upper extremity, which moved synchronously with the movement of the real moving upper extremity (Figure 1), through the HMD, so that they were able to visualize the movement of the left or affected upper extremity continuously. Subjects were asked to perform the wrist flexion-exercise task at a constant pace of 1 Hz, using the right or unaffected upper extremity.

The virtual mirror goal-directed task consisted of catching a cup in the virtual environment by flexing his or her wrist. To reach for the virtual cup, subjects had to flex their virtual wrist until it reached 60°.

### Experiment II(virtual mirror with continuous visual feedback vs intermittent visual feedback experiment)

**Figure 2:**
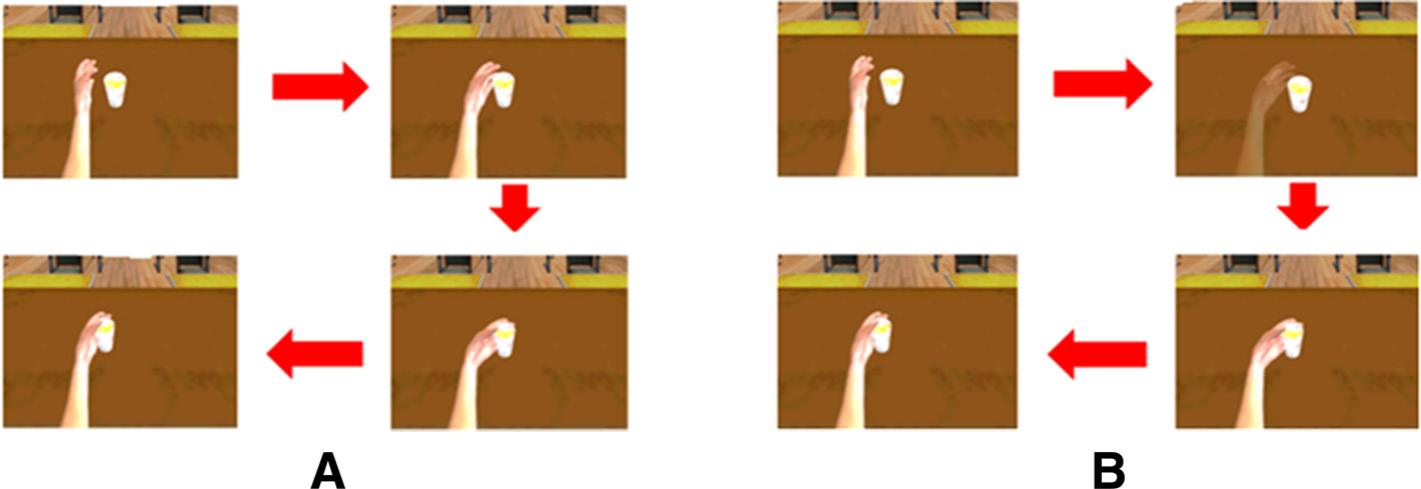
**Setup of the continuous visual feedback (A) and intermittent visual feedback (B) experiments.** During the task (**A**), participants could see a virtual left upper extremity (affected arm in the stroke group) continuously during exercise. However, during task (**B**), the virtual upper extremity and cup became invisible, so that participants had to find the exact position of the virtual cup in the virtual environment using his or her own cognitive domain.

The development process and hardware composition were same in previous virtual mirror experiment. During this experiment, subjects performed three tasks: relaxation and two types of feedback conditions (continuous visual feedback and intermittent visual feedback), in random order. Visual feedback conditions varied in this experiment: One condition consisted of continuously providing visual feedback for the movements and the other consisted of providing visual feedback intermittently, so that subjects had to re-estimate the position of their wrist through the task (Figure 2).

#### Virtual mirror with continuous visual feedback task

The goal-directed task consisted of catching a cup in the virtual environment by flexing his/her wrist. To reach for the virtual cup, subjects had to flex their virtual wrist until it reached 60°, the same as the previous virtual task. Subjects could see the contralateral upper extremity of themselves continuously through the HMD, which moved synchronously with the real moving upper extremity, through the HMD. Subjects performed the exercise as described in the experimental protocol.

#### Virtual mirror with intermittent visual feedback task

In this task, the virtual upper extremity and cup became invisible after for a moment (500 ms) of each trial. Subjects were asked to estimate the position of the cup and flex their wrist to reach the invisible virtual cup. In cases where the wrist position reached to 60° flexion, and could be catch the cup (±3° error was permitted) In cases where the wrist position was incorrect, the position of the virtual cup was shown for a moment (500 ms) and subjects were asked to perform the task again (Figure 2). Twenty MEPs were recorded in the flexion phase of wrist movement corresponding to a wrist joint angle of 5° during every fifth flexion-extension cycle, same in other tasks.

### D. Data analysis

The peak-to-peak amplitude and latency of the MEPs recorded in each condition were measured and averaged to derive mean values. Because the individual mean values of the MEP amplitude were not distributed normally, individual mean values were transformed into the natural logarithm, as suggested by Nielsen [18] for each subject. The individual mean log amplitudes were then entered into a repeated measures one-way analysis of variance (ANOVA). The latency measurement was normally distributed and required no transformation. The Bonferroni multiple comparisons test was also used to compare three conditions which were relaxation, a real mirror task and a virtual mirror task (Experiment I, *P <*0.05). The independent two sample *t*-test was used to perform comparisons which were continuous visual feedback program and intermittent visual feedback program in two groups (Experiment II, *P <*0.05). All data were analyzed using the SPSS software package, version 12.

## Results

### Facilitation of corticospinal excitability during the virtual mirror program in healthy subjects and stroke patients

**Figure 3:**
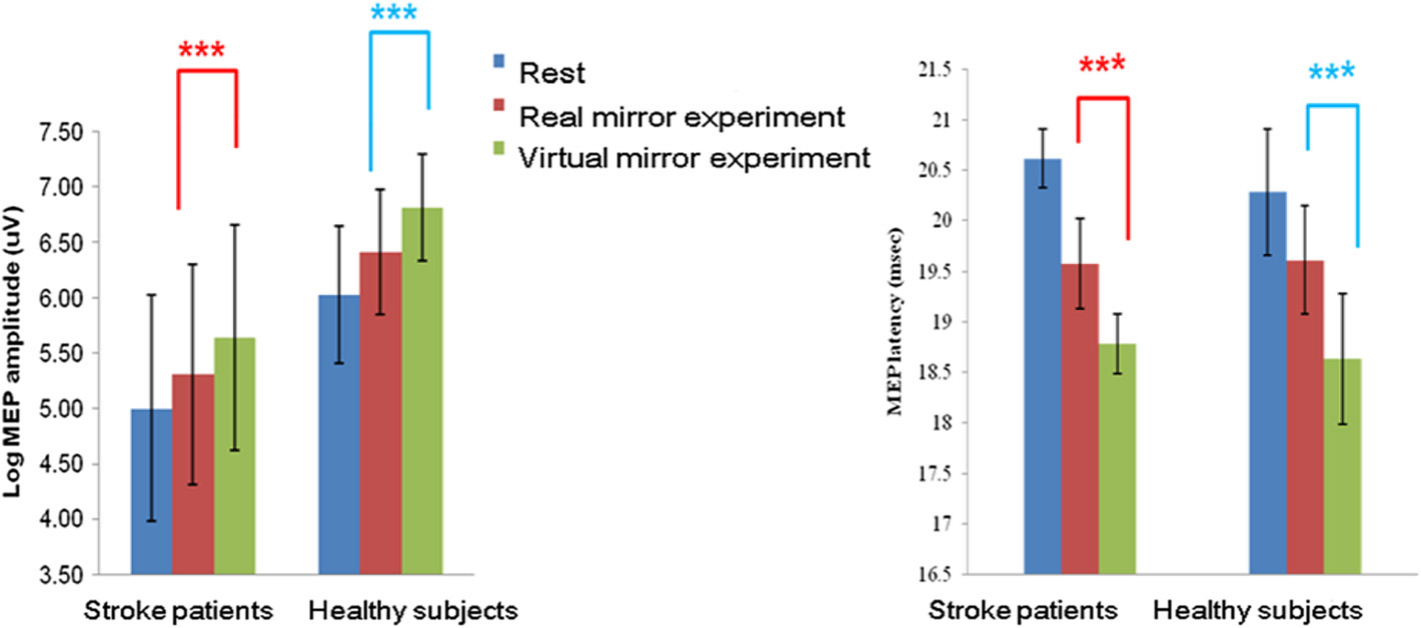
**Facilitation of MEPs during the virtual mirror program in healthy subjects and stroke patients.** The amplitude increment and latency decrement of MEPs in healthy subjects and stroke patients were significantly greater in the virtual mirror paradigm than in the real mirror exercise (repeated measures ANOVA; ***P <0.001).

In healthy subjects, the comparison of log MEP amplitudes across the three tasks revealed a pattern of significant differences (F_2,32_ = 62.2; *P <*0.001; Figure 3). The increment of log mean amplitude and decrement of mean latency of MEPs were significantly greater in the virtual mirror task than in the real mirror task (*P <*0.001; Figure 3). The virtual mirror task increased MEP amplitudes by up to 46.3% (95% CI: 30.4 ~ 80.0) compared with the real mirror task.

In stroke patients, the comparison of MEP amplitudes across the three tasks revealed a pattern of significant differences (F_2, 32_ = 91.9; *P <*0.001; Figure 3). The increment of the mean log amplitude and decrement of mean latency of MEPs were significantly greater in the virtual mirror task than in the real mirror task (*P <*0.001; Figure 3). The virtual mirror program increased MEP amplitudes by up to 44.2% (95% CI: 31.4 ~ 49.9) compared with the real mirror task in stroke patients.

### Facilitation of corticospinal excitability during the visual-feedback-controlled virtual mirror exercise in healthy subjects and stroke patients

**Figure 4:**
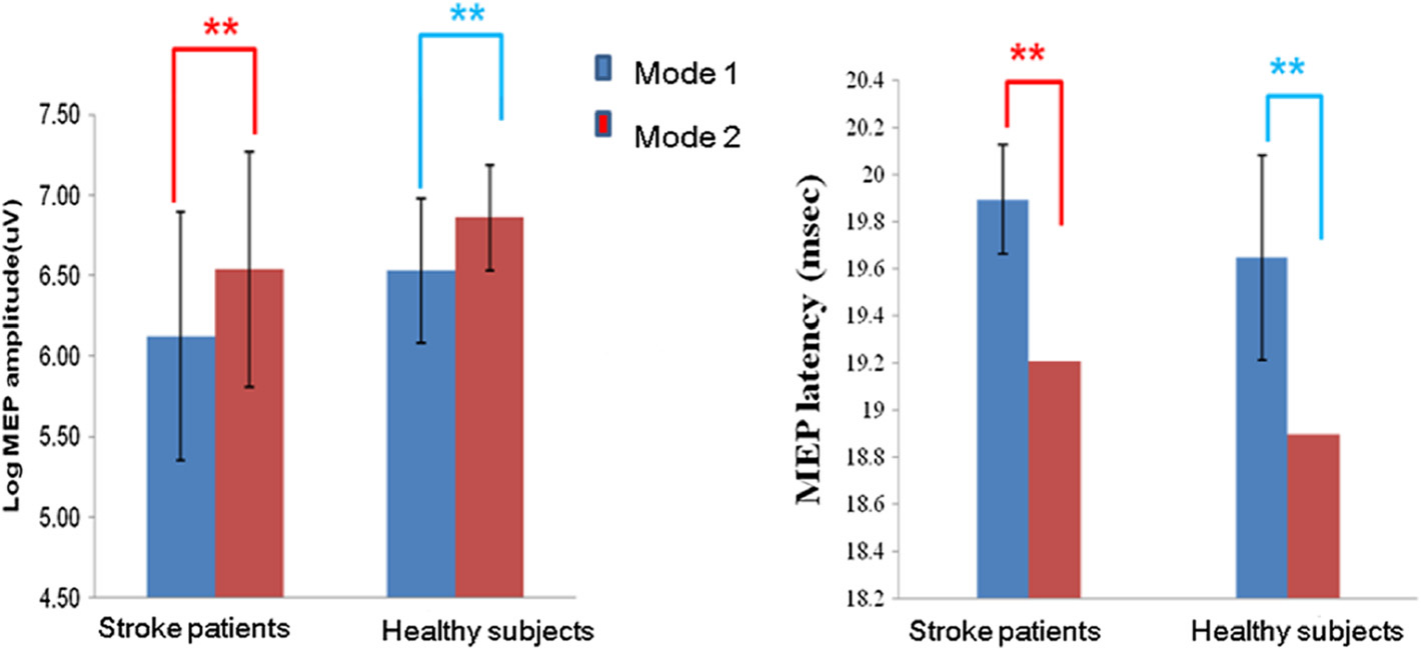
**Facilitation of MEPs during visual-feedback-controlled virtual mirror exercise in healthy subjects and stroke patients.** The amplitude increment and latency decrement of MEPs in healthy subjects and stroke patients were significantly greater for the intermittent visual feedback program (mode 2) than for the continuous visual feedback program(mode 1) (independent two sample *t*-test; **P <0.01).

In healthy subjects, the intermittent visual feedback task yielded higher log MEP amplitudes and lower MEP latency as compared with the continuous visual feedback task (*P <*0.01; Figure 4). The intermittent visual feedback task increased MEP amplitudes by up to 40.4% (95% CI: 26.9 ~ 55.0) compared with the continuous visual feedback task.

A similar result was obtained for stroke patients. The intermittent visual feedback task yielded a higher log MEP amplitude and lower MEP latency as compared to the virtual continuous visual feedback (*P <*0.01; Figure 4). The intermittent visual feedback task using increased MEP amplitudes by up to 48.1% (95% CI: 38.5 ~ 68.7) as compared to the continuous visual feedback task.

## Discussion

In this experiment, both healthy subjects and stroke patients exhibited corticospinal facilitation in conditions of a virtual mirror task and a virtual mirror task with intermittent visual feedback condition.

One explanation for the enhanced facilitation of corticospinal activity by the virtual mirror paradigm compared with the real mirror could be that the virtual mirror paradigm is task oriented (to catch the cup), more interactive and interesting, thus increasing attention and evoking the visual illusion that might activates the putative mirror neuron system and the ipsilesional motor cortex.

It is still a matter of debate whether there is a difference in brain activation between the real and virtual action. Perani et al. found that only real actions in a natural environment activated a visuospatial network in fMRI study [19]. Tai et al. found that the mirror neuronal system was activated in observation of real human grasping task but not in an artificial arm task in PET study [20]. However, Gazzola et al. suggested that a mirror system was strongly activated by the sight of both human and virtual (robotic) actions in fMRI study, with no significant differences. They concluded that the goal might be more important for mirror neuronal activation than the way in which the action is performed [21]. Furthermore, recent TMS research showed that goal-directed movement increases cortical excitability by enhancing the concentration of the patient [22,23].

However, we only recorded the amplitude and latency of MEP (corticospinal excitability) in this study; we assumed that the large amount of facilitation MEP during this study might involve cortical level rather than spinal level. According to a previous study, the large amount of facilitation of MEP during a voluntary contraction of the ipsilateral hand muscle may involve the cortical level rather than spinal level [24]. Furthermore, the facilitation of MEP induced by action observation was attributable to cortico-cortical facilitating connections [25].

The results showing more significant activation of the motor cortex in the intermittent feedback paradigm as compared to the continuous feedback paradigm is intriguing. In the intermittent feedback condition, subjects should continuously predict and move their upper extremity when there is an absence of the virtual upper extremity, which may require an estimation of the target point and induce proprioceptive integrated brain network control. These task-oriented and proprioceptive integrated exercises might activate the extended motor cortex strongly (including the parietal cortex, premotor cortex, and primary motor cortex), which can result in cerebral motor cortical facilitation [26-29].

Although VR has been applied to train and treat stroke patients in order to enhance their abilities (e.g., gait and cognitive ability) [30,31], robust scientific evidence in support of more effective enhancement of motor relearning after stroke or brain damage by VR compared with conventional physical therapy is scarce. In this context, our results of stronger activation of MEPs by the virtual mirror paradigm developed in this study as compared to the real mirror paradigm could be regarded as meaningful. Moreover, the fact that the intermittent visual feedback paradigm evoked stronger MEPs may support evidence that attention to the task, problem-solving ability, and level of difficulty specific to individuals have a considerable effect on motor learning and the contention that the virtual environment may be more beneficial than the real training environment.

An insufficient number of subjects and the heterogeneity of the lesions, onset time, manual dexterity, stage of motor recovery and functional level may represent the limitations of our study. Therefore, we did try to minimize the large variability due to individual difference by being transformed MEP values into natural logarithm, as suggested by Nielsen [18] and the influence of motor recovery on MEP values in the experimental condition by performing the experiment in short period (less than one hour for each subject).

Furthermore, it is hard to generalize this result to the total group of stroke patients, because subjects with better functional ability were recruited. In addition, the lack of assessment of the level of muscle (flexor carpi radialis) contraction during exercise could be another limitation of his study. However, we minimized muscle contraction by placing a roller under the movement plate and by maintaining a constant pace and angle during exercise in each task.

In future investigations, clinical studies examining the effectiveness of the virtual mirror program according to various stroke subgroups, training method, and training duration should be considered, and a method that enhances the effect via integration with other treatment methods, such as robot therapy and the various feedback paradigms, should be developed.

## Conclusion

In both groups, corticospinal excitability was facilitated more in the virtual mirror experiment than in the real mirror experiment. The virtual mirror paradigm with intermittent visual feedback facilitated corticospinal excitability more than the continuous visual feedback, in both groups. This may represent neurophysiological evidence that supports the application of mirror therapy and of the virtual mirror paradigm with various visual modulation technologies to the rehabilitation of upper extremities in stroke patients.

## Abbreviations

VR: Virtual reality; TMS: Transcranial magnetic stimulation; MEP: Motor evoked potential; HMD: Head mounted display.

## Competing interests

No part of this work has been published and no commercial party having a direct financial interest in the results of the research supporting this article has or will confer a benefit on the author(s) or on any organization with which the authors are associated.

## Authors' contributions

YJK conceived of the study, and participated in its design, analysis of results also, write manuscript. HKP carried out the experiment and analysis of the results. HJK participated in its design and coordination and helped to draft the manuscript. SJI carried out the experiment and analysis of the results. JK participated in its design and coordination and helped to draft the manuscript. SC carried out and modulate the experimental setting and performed statistical analysis. SIK conceived of the study, and participated in its design and coordination. ESP conceived of the study, and participated in its design and coordination. All authors read and approved the final manuscript.

## Authors' information

Dr Kang (YJK) is on assistant professor in the Rehabilitation department at Eulji Hospital, Eulji University School of Medicine. She has a Ph.D. degree in Rehabilitation Medicine from Yonsei University of School of Medicine (2011). Her research focuses on rehabilitation of stroke patients using virtual reality or other novel technologies. Prof. Kang has authored recent publications, including “Facilitation of corticospinal excitability according to motor imagery and mirror therapy in healthy subjects and stroke patients (Ann Rehabil Med 2011;35, 747-758)” “Upper Extremity Proprioceptive Assessment Test Using Virtual Environment Technique in Patients with Stroke (J Korean Acad Rehab Med 2010; 34: 141-149)”. “Validity and Reliability of Cognitive Assessment Using Virtual Environment Technology in Patients with Stroke. Am J Phys Med Rehabil 2009;88:702–710”.

## General

Virtual reality (VR) technology has been applied to train and treat stroke patients thereby enhancing their abilities. However, robust scientific evidence supporting greater effective enhancements for motor function relearning after a stroke by VR as compared to conventional physical therapy is scarce. In this context, our results of stronger activation of motor evoked potentials by the virtual mirror paradigm developed in this study compared with the real mirror paradigm could be regarded as meaningful.
